# Maternal folic acid impacts DNA methylation profile in male rat offspring implicated in neurodevelopment and learning/memory abilities

**DOI:** 10.1186/s12263-020-00681-1

**Published:** 2021-01-11

**Authors:** Xinyan Wang, Zhenshu Li, Yun Zhu, Jing Yan, Huan Liu, Guowei Huang, Wen Li

**Affiliations:** 1 Department of Nutrition and Food Science, School of Public Health, Tianjin Medical University, Tianjin, 300070 China; 2 Department of Epidemiology and Biostatistics, School of Public Health, Tianjin Medical University, Tianjin, 300070 China; 3 Tianjin Key Laboratory of Environment, Nutrition and Public Health, Center for International Collaborative Research on Environment, Nutrition and Public Health, Tianjin, 300070 China; 4 Department of Social Medicine and Health Administration, School of Public Health, Tianjin Medical University, Tianjin, 300070 China

**Keywords:** Folic acid, Pregnancy, DNA methylation, Learning/memory, Synaptic plasticity

## Abstract

**Background:**

Periconceptional folic acid (FA) supplementation not only reduces the incidence of neural tube defects, but also improves cognitive performances in offspring. However, the genes or pathways that are epigenetically regulated by FA in neurodevelopment were rarely reported.

**Methods:**

To elucidate the underlying mechanism, the effect of FA on the methylation profiles in brain tissue of male rat offspring was assessed by methylated DNA immunoprecipitation chip. Differentially methylated genes (DMGs) and gene network analysis were identified using DAVID and KEGG pathway analysis.

**Results:**

Compared with the folate-normal diet group, 1939 DMGs were identified in the folate-deficient diet group, and 1498 DMGs were identified in the folate-supplemented diet group, among which 298 DMGs were overlapped. The pathways associated with neurodevelopment and learning/memory abilities were differentially methylated in response to maternal FA intake during pregnancy, and there were some identical and distinctive potential mechanisms under FA deficiency or FA-supplemented conditions.

**Conclusions:**

In conclusion, genes and pathways associated with neurodevelopment and learning/memory abilities were differentially methylated in male rat offspring in response to maternal FA deficiency or supplementation during pregnancy.

## Introduction

Emerging evidence has indicated that early life nutrition influences brain development and exerts long-term consequences for memory abilities profoundly [1, 2]. Periconceptional folic acid (FA) supplementation reduces the incidence of neural tube defects and improves cognitive performances in offspring [3, 4]. Our previous studies demonstrated that maternal FA supplementation during pregnancy promoted offspring’s neurogenesis and synaptogenesis at birth [5], stimulated sensory-motor reflex development in infancy [6, 7], and exerted long-term beneficial effects on learning and memory abilities in adolescence and adult in rat offspring [6]. Our previous study also found maternal folic acid supplementation increased in the offspring’s brains the methylation potential, global DNA methylation level, and DNMT expression and activity [7]. However, the genes or pathways that are epigenetically regulated by FA in neurodevelopment were rarely reported.

Through one-carbon metabolism, folate provides a labile source of methyl groups for numerous methylation reactions, including DNA methylation [8]. DNA methylation and histone modification are the most studied epigenetic modifications reprogrammed considerably in early embryonic development [9]. Epigenetic alterations are thought to module gene expression and silencing [10], and their dysregulation may contribute to numerous neurodevelopment disorders [11, 12]. Maternal FA supplementation during pregnancy increased offspring’s global DNA methylation level in rats [7] and influenced the offspring’s repeat element and imprinted gene methylation in humans [13]. A meta-analysis that included 1988 newborns from two European birth cohorts revealed that maternal plasma folate during pregnancy impacted epigenome-wide DNA methylation in the cord blood. And some of the implicated genes had functional relevance to neurodevelopment [4]. Lambrot et al. [10] provided evidence in mice that lifetime exposure to folate deficiency may cause an altered sperm epigenome and was associated with adverse reproductive outcomes. Transient inhibition of DNA methylation by 5-Azacytidine treatment during development could induce neurodegeneration in neonatal mice and long-lasting deficits in synaptic plasticity and memory abilities in adult mice [14]. Thereby DNA methylation modification may play critical roles in neurodevelopment and learning/memory ability in offspring.

In the present study, male offspring derived from dams fed with FA-deficient, FA-normal, and FA-supplemented diets throughout pregnancy were sacrificed within 24 h after birth to detect DNA methylation profiles. We hypothesized that maternal FA intake during pregnancy might alter DNA methylation profiles established in utero that subsequently regulated neurodevelopment and long-term learning/memory abilities in male offspring.

## Methods

### Rats and dietary treatment

As our previous study [6], 3-month-old female Sprague-Dawley rats (Charles River Laboratories, China) were assigned randomly into three dietary groups (12 rats/group): (1) FA-deficient diet (FA-D), (2) FA-normal diet (FA-N), and (3) FA-supplemented diet (FA-S). Dietary treatment began from 1 week before mating and throughout the end of pregnancy. Rats were housed in a specific pathogen-free facility under a controlled 12-h light/dark cycle and were provided with food and water ad libitum. All animal procedures were approved by the Tianjin Medical University Animal Ethics Committee (TMUaEC2015001).

Diet compositions were modified from the AIN-93G diet (TestDiet, USA), which contains 2.1 mg FA/kg diet. The FA-D and FA-S diets contain 0.1 mg FA/kg diet and 3.5 mg FA/kg diet, respectively. The folate-supplemented diet added a 1.4 mg folic acid/kg diet compared to the folate-normal diet, which in rats is equivalent to consuming a 400 μg folic acid supplement daily on consuming a healthy diet in human. The FA-S group had the highest body weight and brain weight of neonatal offspring; however, there were no differences in brain weight-to-body weight ratio of neonatal offspring within groups. For running methylation analysis, three dams were chosen from each group using random numbers, and then three neonatal male offspring derived from each dam also used random numbers. There were no between-group differences in conception rate, live birth rate, stillbirth rate, absorbed embryo rate, or sex distribution.

The neonatal male offspring were sacrificed within 24 h after birth, and cerebral hemispheres were collected and stored at − 80 °C after liquid nitrogen flash-freezing for further methylated DNA immunoprecipitation (MeDIP) chip assay.

### MeDIP-Chip assay

Genomic DNA was extracted from brain tissue using a DNeasy Blood & Tissue Kit (Qiagen, USA). The purified DNA was quantified using nanodrop ND-1000. Then the genomic DNA was sonicated (Bioruptor sonicator, Diagenode, Belgian) to fragments between 200 and 1000 bp. The MeDIP was prepared as follows: 1 μg of sonicated genomic DNA fragments was denatured for 10 min at 94 °C, immunoprecipitated with mouse anti-5-methylcytosine antibody (1:400, Diagenode, Belgian) overnight at 4 °C, and incubated with anti-mouse IgG magnetic beads for 2 h at 4 °C. After washing, the beads-DNA complex was resuspended in TE buffer with 0.25% SDS and 0.25 mg/mL proteinase K for 2 h at 65 °C. Lastly, the MeDIP DNA was purified using Qiagen MinElute columns (Qiagen, USA).

MeDIP DNA samples and Input samples were labeled with Cy5-9mer and Cy3-9mer primer, respectively, using a NimbleGen Dual-Color DNA Labeling Kit according to the manufacturer’s instruction (Nimblegen Systems, Inc., USA). Then the samples were hybridized to the Arraystar Rat RefSeq Promoter Array, which is designed to investigate the epigenetic modifications and transcription factor binding sites within 15,987 RefSeq Gene promoter regions covered by approximately 180,000 probes. Scanning was performed with the Agilent Scanner G2505C microarray scanner.

### Identification of differentially methylated regions

The log_2_ (MeDIP/Input) getting from raw data was normalized using the Median-centering, quantile normalization, and linear smoothing analysis by Bioconductor packages Ringo, limma, and MEDME. Then from the normalized log_2_ (MeDIP/Input) data, a sliding-window peak-finding algorithm provided by NimbleScan v2.5 (Roche-NimbleGen) was applied to find the enriched peaks with specified parameters (sliding window width 1500 bp; minimum probes per peak 2; *P* value minimum cut-off (-log10) 2), and peaks within 500 bp spacing were merged.

To compare the differentially enriched regions between two groups, the log_2_ (MeDIP/Input) was averaged for each group (experiment and control), and the *M’* value for each probe was calculated as follows:

*M’* = Average (log_2_MeDIP_E_/Input_E_) − Average (log_2_MeDIP_C_/Input_C_)

Then the NimbleScan sliding-window peak-finding algorithm was rerun on these data to find the differential enrichment peaks (DEPs) between groups, filtered according to the following criteria:

1. At least one of the two groups had a median (log_2_ MeDIP/Input) ≥ 0.3 and a median (*M'*) > 0

2. At least half of probes in a peak might have a coefficient of variability ≤ 0.8 in both groups

### Promoter classification

Promoters were defined as the regions [− 1300 bp, + 500 bp] around the transcription start sites (TSS). Promoters were classified into 3 categories, as follows:

1. High-CpG-density promoter (HCP): promoters containing a 500-bp window with GC content ≥ 55%, and CpG observed to an expected ratio (O/E) ≥ 0.6 within − 700 to + 200 bp around TSS

2. Low-CpG-density promoter (LCP): promoters containing no 500-bp interval and a CpG O/E ≥ 0.4

3. Intermediate-CpG-density promoter (ICP): remaining promoters that were neither HCP nor LCP

### Functional analysis

Genes containing DEPs in the promoter were considered as the differentially methylated genes (DMGs). Enrichments in the biological process of gene ontology (GO) and pathways that have functional relevance to neurodevelopment and learning/memory abilities were analyzed using DAVID Bioinformatics Resources 6.8 [15] and KEGG pathway analysis [16], respectively.

## Results

### Maternal FA intake during pregnancy altered DNA methylation profiles in the brain tissue of male offspring

To investigate the DNA methylation profiles altered by maternal FA intake during pregnancy, DEPs in the promoter region between groups were identified using the NimbleScan v2.5 software (Roche-NimbleGen). Compared with the FA-N group, 1843 DEPs were identified in the FA-D group (744 hypermethylated and 1099 hypomethylated), and 1406 DEPs were identified in the FA-S group (738 hypermethylated and 668 hypomethylated).

Since one single peak can be assigned to more than one gene promoter, and one promoter may contain more than one peak, the peaks-to-promoters are not always one-to-one correspondence. The DEPs were further analyzed according to their assigned gene promoter. Comparing the FA-D group with the FA-N group (FA-D/FA-N), there are 996, 438, and 433 DEPs located in HCP, ICP, and LCP, respectively, while there were 699, 392, and 337 DEPs located, representatively, in the HCP, ICP, and LCP in the FA-S group compared with the FA-N group (FA-S/FA-N). Those DEPs constituted 1939 DMGs in FA-D/FA-N, and 1498 DMGs in FA-S/FA-N, among which 298 DMGs were overlapped.

Taken together, the results indicated that maternal FA intake during pregnancy altered DNA methylation profiles in brain tissue of neonatal male offspring, and DEPs localized mostly (approximately 50%) in HCP.

### Maternal FA intake during pregnancy altered biological process involved in neurodevelopment and learning/memory abilities in the brain tissue of male offspring

We identified some identical and distinctive DMGs altered in response to maternal FA deficiency and supplementation during pregnancy. For this reason, data of the maternal FA deficiency (FA-D) or supplementation group (FA-S) are compared with the FA normal group (FA-N) separately. GO analysis revealed that DMGs in FA-D/FA-N were enriched in a wide variety of biological processes involved in neural tube development, neural crest cell migration, brain development, neuron differentiation, glia cell differentiation, synapse organization, chemical synaptic transmission, and learning/memory (Table 1). And DMGs in FA-S/FA-N were enriched in biological processes involved in neural tube development, brain development, neuron differentiation, synapse organization, myelination, chemical synaptic transmission, and memory (Table 2).

**Table 1:** Gene implicated in neurodevelopment and learning/memory abilities were differentially methylated in brain tissue of FA-D male offspring compared with the FA-N male offspring

GO term (BP)	DMGs associated
Dorsal/ventral neural tube patterning	5 genes: Psen1 (hypermethylated), Gsc, Kif3a, Pax7, Ptch1 (hypomethylated)
Neural crest cell migration	14 genes: Pax6, Coro1c, Sema5a, Sema6d, Sema3b (hypermethylated), Ret, Efnb1, Kitlg, Isl1, Sox8, Sema6c, Sema7a, Gbx2, Sema4b (hypomethylated)
Brain development, hippocampus development, orbitofrontal cortex development, cerebellum development	59 genes: Fgfr1, Stk36, Hmgcs1, Pax6, Ptprs, Cdk5, Tp73, Bbs2, Casp3, Acat2, Cttnbp2, Ace, Ndrg4, Synj2, Ntf3, Gsx2, Ak2, Ager, Notch1, Psen1, Snrpb, Pygo2 (hypermethylated), Cast, Pfkfb3, Phlpp2, Lmx1a, Ogdh, Igf1r, Atp2b4, Akap5, Dclk2, Lhx5, Reln, Pafah1b1, Rara, Eif2b4, Fgfr2, Sstr1, Glud1, Gbx2, Sdf4, Rere, Dmrta2, Tmem57, Cxcr4, Sharpin, Unc5c, Irs2, Col4a1, Mdga1, Gabra5, Dpysl2, Pcdh19, Ddit4, Ptpn11, Hes5, Abcb1b, Ptch1, Cln5 (hypomethylated)
Neuron differentiation, neuron projection development, positive regulation of neuron projection development, negative regulation of neuron projection development, axonogenesis, negative regulation of axonogenesis, axon guidance, negative regulation of axon extension involved in axon guidance, axon regeneration, negative regulation of axon regeneration, negative regulation of dendrite development	112 genes: Wnt10a, Wnt5b, Cebpb, Fgfr1, Ncdn, Ager, Cdk5, Wnt7b, Cd44, Prkci, Kidins220, Fam134c, Nme2, Ndrg4, Ntrk1, Neu1, Tmem30a, Lrp1, Actb, Erbb3, Lrtm2, Flot1, Pax6, Slitrk2, Notch1, Gas7, Casp3, Sema5a, Sema6d, Sema3b, Dhfr, Folr1, Klk8, Fat3, Cyth2, Bag1, Psen1, Mbp, Ntf3, Kif5a, Etv1, B3gnt2 (hypermethylated), Ret, Sox11, Neurog1, Pld2, Ptprm, Vapa, Rb1, Itgb1, Pten, Uhmk1, Igf1r, Lingo1, App, Sema7a, Camsap1, Map4, Runx3, Arsb, Ltk, Katnb1, Ankrd1, Kif3c, Bcl11a, Ntrk2, Adamts1, Reln, Ngfr, Scarb2, Bag5, Mag, Fkbp4, Adcy5, Cspg4, Lrig2, Pafah1b1, Paqr3, Fgfr2, Adcy1, Atl1, Stk11, Nkx2-8, Lrrn3, Rtn4r, Ptpn11, Picalm, Lmx1a, Isl1, Ddit4, Wnt3, Kdm2a, Sema6c, Sema4b, Jak2, Ctnna1, Epha4, Arf6, Hes5, Ppia, Acsl4, Runx2, Wnt7a, Lrp4, Efna2, Efnb1, Dpysl2, Ttc8, Gbx2, Unc5c, Kif26a, Kif26b (hypomethylated)
Astrocyte differentiation, oligodendrocyte differentiation	11 genes: Notch1, S100b, Pax6, Bnip3, Cdk5 (hypermethylated), Hes5, Ptprj, Ntrk2, Olig1, Sox8, Nkx6-1 (hypomethylated)
Synapse organization	7 genes: Sncg, Wnt7b, Klk8 (hypermethylated), LRRTM1, Lmx1a, Wnt7a, Lrp4 (hypomethylated)
Synaptic transmission, glutamatergic, positive regulation of synaptic transmission, glutamatergic	13 genes: P2rx1, Cnih2, Grin2d, Grin1, Cdk5, Egfr, Cckbr, Ntrk1 (hypermethylated), Clcn3, Unc13b, Ntrk2, Reln, Ngfr (hypomethylated)
Learning or memory, memory, associative learning	29 genes: Egfr, Casp3, Sts, Psen1, S100b, Dyx1c1, Prkar1b, Ntrk1, Grin1, Abca7, Klk8, Cebpb, Paip2, Th, Nrgn, Cdk5 (hypermethylated), Pafah1b1, Pten, Igf2, Lmx1a, Itpr3, Sorcs3, Adrb1, Plk2, Reln, Chrnb2, Cacna1d, Gabra5, Neurod2 (hypomethylated)

The MeDIP-Chip assay was performed on brain tissue from folic acid-deficient (FA-D) and folic acid-normal (FA-N) male offspring (*n* = 3/group). Enriched biological processes (BP) of GO terms by DAVID were used to identify the differentially methylated genes (DMGs) associated with neurodevelopment and learning/memory abilities

**Table 2:** Gene implicated in neurodevelopment and learning/memory abilities were differentially methylated in brain tissue of FA-S male offspring compared with the FA-N male offspring

GO term (BP)	DMGs associated
Neural fold formation	3 genes: Nodal, Cfl1 (hypermethylated), Cecr2 (hypomethylated)
Forebrain development, lateral ventricle development, hindbrain development	16 genes: Dlc1, App, Kdm2b, Sstr1, Neurog3, Hspa8, Aqp1, Dbi, Hoxa1, Neurod1 (hypermethylated), Dkk1, Htra2, Zic5, Apaf1, Smarca4, Tsku (hypomethylated)
Neuron projection development	16 genes: Stx3, Clmn, Itgb1, Pten, App, Npy, Cdnf, Sema7a, Ehd1 (hypermethylated), Ptprm, Ncdn, Camsap1, Shc1, Areg, Ikbkb, Runx3 (hypomethylated)
Synapse maturation	3 genes: Pten (hypermethylated), Erbb4, Shank1 (hypomethylated)
Myelination	9 genes: Dhh, Nab1, Afg3l2 (hypermethylated), Amigo1, Plp1, Nab2, Nfasc, Olig2, Tgfb1 (hypomethylated)
Positive regulation of synaptic transmission, GABAergic, synaptic vesicle docking, long-term synaptic potentiation, long term synaptic depression	15 genes: Tacr1, Tac1, Stx3, Syde1, Plk2, Dbi, Pten, Drd5 (hypermethylated), Erbb4, Prkce, Car7, Stx4, Slc8a2, S100b, Shank2 (hypomethylated)
Memory	11 genes: Pak6, Plk2, Slc6a4, Pten (hypermethylated), Slc8a2, Cebpb, Syt4, S100b, Cacna1d, Shank2, Neto1 (hypomethylated)

The MeDIP-Chip assay was performed on brain tissue from folic acid-supplemented (FA-S) and folic acid-normal (FA-N) male offspring (*n* = 3/group). Enriched BP of GO terms by DAVID were used to identify the DMGs associated with neurodevelopment and learning/memory abilities

Although some biological process involved in neurodevelopment and learning/memory abilities are shared by different FA status, compared with FA-S/FA-N, more DMGs implicated in brain development and neuron differentiation were found in FA-D/FA-N. Additionally, DMGs in FA-D/FA-N are distinctively enriched in neural crest cell migration and glia cell differentiation, while DMGs in FA-S/FA-N are distinctively enriched in myelination (Tables 1 and 2).

Taken together, the results indicated that genes implicated in neurodevelopment and learning/memory abilities were differentially methylated in response to maternal FA intake during pregnancy, and there were some identical and distinctive biological processes altered under FA deficiency and FA-supplemented conditions.

### Maternal FA intake during pregnancy impacting pathways implicated in the neurodevelopment of male offspring

To further discover the potential mechanisms whereby maternal FA intake during pregnancy impacts neurodevelopment through DNA methylation, KEGG pathway analysis was performed on DMGs to identify the enriched pathways associated with neurodevelopment in FA-D/FA-N and FA-S/FA-N. The results showed that DMGs altered by maternal FA deficiency were enriched in pathways including signaling pathways regulating pluripotency of stem cells, autophagy, tight junction, calcium signaling pathway, mammalian/mechanistic target of rapamycin (mTOR) signaling pathway, Notch signaling pathway, neurotrophin signaling pathway, and protein processing in the endoplasmic reticulum (ER) (FA-D/FA-N in Table 3). In contrast, DMGs altered by maternal FA supplementation were enriched in pathways including signaling pathways regulating pluripotency of stem cells, autophagy, tight junction, calcium signaling pathway, mitogen-activated protein kinase (MAPK) signaling pathway, transforming growth factor-beta (TGF-β) signaling pathway, and axon guidance (FA-S/FA-N in Table 3).

**Table 3:** Pathways associated with neurodevelopment were differentially methylated in the brain of males in response to maternal FA intake during pregnancy

Group/pathway	No of DMGs (%)^**a**^	Selected genes	***P*** value
**FA-D/FA-N**			
Signaling pathways regulating pluripotency of stem cells^b^	25/139 (17.99)	**Wnt10a**, Fgfr1, Mapk12, Pax6, Smad1, Wnt5b, Wnt7b (hypermethylated), **Isl1**, **Neurog1**, **Onecut1**, Bmpr1b, Dvl3, Esrrb, Fgfr2, Igf1r, Jak2, Klf4, Lhx5, Nanog, Pcgf1, Smad2, Smad3, Tbx3, Wnt3, Wnt7a (hypomethylated)	1.05E−03
Autophagy—animal^b^	23/134 (17.16)	Bnip3, Mtmr4, Rraga (hypermethylated), **Irs2**, **Irs3**, **Nrbf2**, **Pten**, **Rb1cc1**, **Rragd**, **Ulk2**, Atg10, Bad, Ddit4, Eif2ak3, Ern1, Igf1r, Irs1, Lamp1, Ppp2cb, Prkaa1, Stk11, Stx17, Uvrag (hypomethylated)	3.07E−03
Tight junction^b^	25/170 (14.71)	**Nedd4l**, Actb, Arhgap17, Cldn18, Dlg2, Marveld3, Mpp4, Myh7b, Myl9, Prkag1, Prkci, Rapgef6 (hypermethylated), **Cacna1d**, **Cldn17**, **Ezr**, **Itgb1**, **Slc9a3r1**, Crb3, F11r, Hspa4, Marveld2, Ppp2cb, Prkaa1, Prkag2, Stk11 (hypomethylated)	1.54E−02
Calcium signaling pathway^b^	27/188 (14.36)	**Grin2d**, Adcy4, Calml3, Cckbr, Ednra, Egfr, Erbb3, Grin1, Mylk2, P2rx1, Ptger1, Tnnc2, Trhr (hypermethylated), **Adcy3**, **Adcy7**, **Cacna1d**, **Cacna1h**, **Gnal**, **Phkg2**, Adcy1, Adrb1, Atp2b4, Gna11, Itpr3, Plcd3, Stim2, Vdac3 (hypomethylated)	1.63E−02
mTOR signaling pathway	26/157 (16.56)	Atp6v1f, Flcn, Rps6ka1, Rraga, Slc3a2, Wnt10a, Wnt5b, Wnt7b (hypermethylated), Atp6v1b2, Atp6v1e2, Atp6v1h, Cab39, Ddit4, Dvl3, Eif4e2, Igf1r, Irs1, Prkaa1, Pten, Rps6ka3, Rragd, Sos1, Stk11, Ulk2, Wnt3, Wnt7a (hypomethylated)	2.86E−03
Notch signaling pathway	10/52 (19.23)	Aph1b, Notch1, Psen1 (hypermethylated), Ctbp2, Dtx3l, Dvl3, Hes5, Jag1, Lfng, Notch2 (hypomethylated)	2.09E−02
Neurotrophin signaling pathway	19/124 (15.32)	Calml3, Irak3, Irak4, Kidins220, Mapk12, Ntf3, Ntrk1, Psen1, Rps6ka1, Tp73 (hypermethylated), Arhgdia, Bad, Irs1, Ngfr, Ntrk2, Ptpn11, Rela, Rps6ka3, Sos1 (hypomethylated)	2.17E−02
Protein processing in endoplasmic reticulum	23/164 (14.02)	Bag1, Ddit3, Dnajc5b, Gcs1, Hsph1, Nploc4, Preb, Stt3a (hypermethylated), Ckap4, Derl1, Dnaja2, Eif2ak3, Ern1, P4hb, Rad23b, Sec31a, Sec61a1, Sec61a2, Ssr1, Stt3b, Stub1, Ubqln1, Yod1 (hypomethylated)	3.23E−02
**FA-S/FA-N**			
Signaling pathways regulating pluripotency of stem cells^b^	18/139 (12.95)	**Neurog1**, Dusp9, Hnf1a, Hras, Inhbb, Inhbe, Mapk3, Myc, Nodal, Pcgf3, Pcgf5, Zic3 (hypermethylated), **Isl1**, **Onecut1**, **Wnt10a,** Bmpr2, Fzd4, Map2k2 (hypomethylated)	8.27E−03
Autophagy—animal^b^	18/134 (13.43)	**Irs2**, **Pten**, **Rb1cc1**, Atg2a, Atg3, Hras, Mapk3, Rps6kb1, RragB (hypermethylated), **Irs3**, **Nrbf2**, **Rragd**, **Ulk2,** Atg9b, Eif2s1, Gabarapl2, Map2k2, Rps6kb2 (hypomethylated)	5.67E−03
Tight junction^b^	21/170 (12.35)	**Cldn17**, **Itgb1**, Actr3b, Bves, Sympk, Tuba3b, Tuba4a (hypermethylated), **Cacna1d**, **Ezr**, **Nedd4l**, **Slc9a3r1**,Cldn11, Cldn19, Cttn, Gata4, Map3k1, Myl2, Ppp2r1b, Prkag3, Prkce, Synpo (hypomethylated)	7.82E−03
Calcium signaling pathway^b^	27/188 (14.36)	**Adcy3**, **Adcy7**, **Cacna1h**, **Phkg2**, Calm3, Camk2a, Drd5, Grpr, Htr2c, P2rx4, P2rx6, Phka2, Ppp3r2, Tacr1 (hypermethylated), **Cacna1d**, **Gnal**, **Grin2d**, Adcy2, Camk2d, Erbb4, Gna14, Htr5b, Ltb4r2, Mylk, Nos3, P2rx5, Slc8a2 (hypomethylated)	2.73E−04
MAPK signaling pathway	37/298 (12.42)	Cacna1h, Dusp4, Dusp8, Dusp9, Fgf16, Fgf4, Hras, Hspa8, Hspb1, Il1rap, Map4k3, Mapk3, Mapk7, Myc, Myd88, Pla2g4b, Ppp3r2, Rac2 (hypermethylated), Cacna1d, Cacng6, Cdc25b, Dusp3, Fas, Fgf20, Ikbkb, Il1r1, Kitlg, Map2k2, Map3k1, Map3k12, Map3k13, Map4k4, Mapk8ip1, Tgfa, Tgfb1, Traf2, Vegfa (hypomethylated)	4.51E−04
TGF-beta signaling pathway	13/87 (14.94)	Cdkn2b, Fst, Inhbb, Inhbe, Ltbp1, Mapk3, Myc, Nodal, Rps6kb1 (hypermethylated), Bmpr2, Ppp2r1b, Rps6kb2, Tgfb1 (hypomethylated)	7.30E−03
Axon guidance	19/178 (10.67)	Camk2a, Cfl1, Dpysl5, Hras, Itgb1, Mapk3, Nfatc4, Pak6, Plxnb3, Ppp3r2, Rac2, Sema4a, Sema7a, Trpc3 (hypermethylated), Bmpr2, Camk2d, Ntng1, Rgs3, Srgap2 (hypomethylated)	4.35E−02

The genes which altered methylation in both FA-D and FA-S were showed by bold type. The *P* value was calculated by Fisher’s exact test

^a^The ratio of no. of DMGs was calculated by the number of DMGs in a given pathway divided by the total number of genes in this pathway

^b^The pathways overlapped between FA-D/FA-N and FA-S/FA-N

The results showed that signaling pathways regulating pluripotency of stem cells, autophagy, tight junction, and calcium signaling pathway were overlapped between FA-D/FA-N and FA-S/FA-N, indicating the mechanisms that shared from the FA-deficient status to the FA-normal status and to the FA-supplemented status. Additionally, DMGs in FA-D/FA-N enriched distinctively in mTOR signaling pathway, Notch signaling pathway, neurotrophin signaling pathway, and protein processing in ER, while DMGs in FA-S/FA-N enriched distinctively in MAPK signaling pathway, TGF-β signaling pathway, and axon guidance (Table 3), indicating distinctive mechanisms of influencing neurodevelopment in male offspring under FA-deficient or FA-supplemented conditions.

### Maternal FA intake during pregnancy impacting pathways implicated in learning/memory abilities of male offspring

To illustrate the potential mechanisms of maternal FA during pregnancy impacting offspring’s learning/memory abilities by DNA methylation, enriched pathways associated with synaptic plasticity and learning/memory abilities were identified using KEGG pathway analysis. The results showed that DMGs altered by maternal FA deficiency were enriched in pathways including cGMP-PKG signaling pathway, gap junction, calcium signaling pathway, mTOR signaling pathway, Rap1 signaling pathway, Notch signaling pathway, endocytosis, and neurotrophin signaling pathway (FA-D/FA-N in Table 4), while DMGs altered by maternal FA supplementation were enriched in pathways including cGMP-PKG signaling pathway, gap junction, calcium signaling pathway, MAPK signaling pathway, ErbB signaling pathway, glutamatergic synapse, and neuroactive ligand-receptor interaction (FA-S/FA-N in Table 4).

**Table 4:** Pathways associated with learning/memory abilities were differentially methylated in the brain of males in response to maternal FA intake during pregnancy

Group/pathway	No. of DMGs (%)^**a**^	Selected genes	***P*** value
**FA-D/FA-N**			
cGMP-PKG signaling pathway^b^	29/167 (17.37)	**Gucy1a2**, **Gucy1a3**, Adcy4, Calml3, Ednra, Myh7, Myl9, Mylk2, Pde2a (hypermethylated), **Adcy3**, **Adcy7**, **Cacna1d**, **Irs2**, **Irs3**, Adcy1, Adcy5, Adrb1, Atp2b4, Bad, Gna11, Gna13, Irs1, Itpr3, Kcnmb4, Mrvi1, Pde2a, Pde3b, Pde5a, Ppp1ca, Vdac3 (hypomethylated)	7.83E−04
Gap junction^b^	15/88 (17.05)	**Gucy1a2**, **Gucy1a3,** Adcy4, Egfr (hypermethylated), **Adcy3**, **Adcy7**, Adcy1, Adcy5, Adrb1, Cdk1, Gna11, Itpr3, Pdgfc, Sos1, Tubb6 (hypomethylated)	1.61E−02
Calcium signaling pathway^b^	27/188 (14.36)	**Grin2d**, Adcy4, Calml3, Cckbr, Ednra, Egfr, Erbb3, Grin1, Mylk2, P2rx1, Ptger1, Tnnc2, Trhr (hypermethylated), **Adcy3**, **Adcy7**, **Cacna1d**, **Cacna1h**, **Gnal**, **Phkg2**, Adcy1, Adrb1, Atp2b4, Gna11, Itpr3, Plcd3, Stim2, Vdac3 (hypomethylated)	1.63E−02
mTOR signaling pathway	26/157 (16.56)	Atp6v1f, Flcn, Rps6ka1, Rraga, Slc3a2, Wnt10a, Wnt5b, Wnt7b (hypermethylated), Atp6v1b2, Atp6v1e2, Atp6v1h, Cab39, Ddit4, Dvl3, Eif4e2, Igf1r, Irs1, Prkaa1, Pten, Rps6ka3, Rragd, Sos1, Stk11, Ulk2, Wnt3, Wnt7a (hypomethylated)	2.86E−03
Rap1 signaling pathway	30/217 (13.82)	Actb, Adcy4, Calml3, Egfr, Fgf14, Fgf21, Fgfr1, Grin1, Mapk12, Prkci, Rapgef6, Skap1, Vegfb (hypermethylated), Adcy1, Adcy3, Adcy5, Adcy7, Efna2, Fgf12, Fgf20, Fgfr2, Igf1r, Itgb1, Kitlg, Map2k3, Ngfr, Pdgfc, Pfn1, Rasgrp3, Sipa1l2 (hypomethylated)	1.93E−02
Notch signaling pathway	10/52 (19.23)	Aph1b, Notch1, Psen1 (hypermethylated), Ctbp2, Dtx3l, Dvl3, Hes5, Jag1, Lfng, Notch2 (hypomethylated)	2.09E−02
Endocytosis	35/263 (13.31)	Ap2m1, Arpc4, Chmp1a, Chmp2a, Chmp5, Cyth2, Dnm3, Egfr, Folr1, Gbf1, Grk5, Iqsec3, Kif5a, Nedd4l, Prkci, RT1-M10-1, Sh3kbp1, Stam2, Stambp (hypermethylated), Arf1, Arf5, Arf6, Capza2, Cxcr4, Fgfr2, Git2, Igf1r, Pld2, Rab11fip1, Sh3glb2, Smad2, Smad3, Snx2, Spg20, Vps37a (hypomethylated)	2.09E−02
Neurotrophin signaling pathway	19/124 (15.32)	Calml3, Irak3, Irak4, Kidins220, Mapk12, Ntf3, Ntrk1, Psen1, Rps6ka1, Tp73 (hypermethylated), Arhgdia, Bad, Irs1, Ngfr, Ntrk2, Ptpn11, Rela, Rps6ka3, Sos1 (hypomethylated)	2.17E−02
**FA-S/FA-N**			
cGMP-PKG signaling pathway^b^	25/167 (14.97)	**Adcy3**, **Adcy7**, **Gucy1a2**, **Irs2**, Calm3, Cngb1, Mapk3, Nfatc4, Ppp3r2 (hypermethylated), **Cacna1d**, **Gucy1a3**, **Irs3**, Adcy2, Adcy6, Adra2c, Atp1b1, Atp1b3, Fxyd2, Gata4, Kcnma1, Map2k2, Mylk, Nos3, Prkce, Slc8a2 (hypomethylated)	2.38E−04
Gap junction^b^	14/88 (15.91)	**Adcy3**, **Adcy7**, **Gucy1a2**, Csnk1d, Hras, Htr2c, Mapk3, Mapk7, Tuba3b, Tuba4a (hypermethylated), **Gucy1a3**, Adcy2, Adcy6, Map2k2 (hypomethylated)	3.07E−03
Calcium signaling pathway^b^	27/188 (14.36)	**Adcy3**, **Adcy7**, **Cacna1h**, **Phkg2**, Calm3, Camk2a, Drd5, Grpr, Htr2c, P2rx4, P2rx6, Phka2, Ppp3r2, Tacr1 (hypermethylated), **Cacna1d**, **Gnal**, **Grin2d**, Adcy2, Camk2d, Erbb4, Gna14, Htr5b, Ltb4r2, Mylk, Nos3, P2rx5, Slc8a2 (hypomethylated)	2.73E−04
MAPK signaling pathway	37/298 (12.42)	Cacna1h, Dusp4, Dusp8, Dusp9, Fgf16, Fgf4, Hras, Hspa8, Hspb1, Il1rap, Map4k3, Mapk3, Mapk7, Myc, Myd88, Pla2g4b, Ppp3r2, Rac2 (hypermethylated), Cacna1d, Cacng6, Cdc25b, Dusp3, Fas, Fgf20, Ikbkb, Il1r1, Kitlg, Map2k2, Map3k1, Map3k12, Map3k13, Map4k4, Mapk8ip1, Tgfa, Tgfb1, Traf2, Vegfa (hypomethylated)	4.51E−04
ErbB signaling pathway	15/89 (16.85)	Camk2a, Hras, Mapk3, Myc, Pak6, Rps6kb1, Shc2 (hypermethylated), Areg, Camk2d, Erbb4, Map2k2, Rps6kb2, Shc1, Shc3, Tgfa (hypomethylated)	1.22E−03
Glutamatergic synapse	14/116 (12.07)	Adcy3, Adcy7, Grm6, Mapk3, Pla2g4b, Ppp3r2, Slc17a8, Slc38a1 (hypermethylated), Adcy2, Adcy6, Cacna1d, Grin2d, Shank1, Shank2 (hypomethylated)	3.20E−02
Neuroactive ligand-receptor interaction	29/291 (9.97)	Chrna2, Drd5, Gabra5, Gabrd, Gabre, Gcgr, Gpr50, Grm6, Grpr, Htr1a, Htr2c, P2rx4, P2rx6, Prss2, Pth1r, Sstr1, Tacr1, Uts2r (hypermethylated), Adra2c, Chrna1, Glp2r, Grid1, Grin2d, Htr5b, Ltb4r2, Mc2r, P2rx5, Prlhr, Ptgir (hypomethylated)	3.44E−02

The genes which altered methylation in both FA-D and FA-S were showed by bold type. The *P* value was calculated by Fisher’s exact test

^a^The ratio of No. of DMGs was calculated by the number of DMGs in a given pathway divided by the total number of genes in this pathway

^b^The pathways overlapped between FA-D/FA-N and FA-S/FA-N groups

The results showed that cGMP-PKG signaling pathway, gap junction, and calcium signaling pathway were overlapped between FA-D/FA-N and FA-S/FA-N, indicating the mechanisms that were shared from the FA-deficient status to the FA-normal status and to the FA-supplemented status. Additionally, DMGs in FA-D/FA-N enriched distinctively in mTOR signaling pathway, Rap1 signaling pathway, Notch signaling pathway, endocytosis, and neurotrophin signaling pathway, while DMGs in FA-S/FA-N enriched distinctively in MAPK signaling pathway, ErbB signaling pathway, glutamatergic synapse, and neuroactive ligand-receptor interaction (Table 4), indicating the distinctive mechanisms of influencing learning/memory abilities in male offspring under maternal FA-deficient or FA-supplemented conditions.

## Discussion

In the present study, the results showed that maternal FA intake during pregnancy altered DNA methylation profiles in brain tissue of neonatal male offspring, and pathways associated with neurodevelopment and learning/memory were differentially methylated. DMGs altered by maternal FA deficiency (FA-D/FA-N) were enriched in eight pathways associated with neurodevelopment and eight pathways implicated in learning/memory abilities, while DMGs altered by maternal FA supplementation (FA-S/FA-N) were enriched in seven pathways implicated in neurodevelopment and seven pathways implicated in learning/memory abilities. There were some identical and distinctive potential mechanisms impacting neurodevelopment or learning/memory abilities under FA deficiency or FA-supplemented conditions.

Folate is an essential micronutrient required for cellular proliferation through de novo nucleotide synthesis and epigenetic regulation of gene expression through methylation. As others and our previous study, deficient or folic acid supply during pregnancy alter cortical neurodevelopment and behavior in rodents [5–7, 17]. During pregnancy, folate deficiency results in a persistent and comparable deviation in cortical cytoarchitectural organization, and underperform in behavior development [6, 7, 17]. During pregnancy, the recommended supplement daily intake of folic acid promoted offspring’s neurogenesis and synaptogenesis at birth [5] and stimulated sensory-motor reflex development in infancy [6, 7]. But excess folic acid supply (10 times of folate-normal diet) during pregnancy is also bad for cortical neurodevelopment and behavior in the early postnatal stage [17]. And our previous study also found that maternal folic acid supplementation increased in offspring’s brains the methylation potential, global DNA methylation level, and DNMT expression and activity [7]. Excess folic acid supply (10 times of folate-normal diet) during pregnancy changed folate profile but did not alter the global DNA methylation in brain tissue [17].

### Potential mechanisms of maternal FA intake during pregnancy impacting offspring’s neurodevelopment through DNA methylation

DNA methyltransferases (DNMT) 3a-null mice showed impairments in postnatal neurogenesis [18], and DNMT3b knockdown in human embryonic stem cells altered the timing of neuronal differentiation and maturation [19], supporting vital roles of DNA methylation in neurodevelopment. Consistently, the results in the present study illustrated that the DMGs altered by maternal FA deficiency and supplementation were both enriched in pathways of signaling pathways regulating pluripotency of stem cells, autophagy, tight junction, and calcium signaling pathway, all of which are associated with neurodevelopment. Signaling pathways regulating pluripotency of stem cells activate a core transcriptional network, including Oct4, Nanog, and Sox2. These three transcription factors coordinate with their downstream target genes to promote self-renewal and pluripotency of stem cells, thereby impacting embryonic development and neurodevelopment. Autophagy, a self-degradative process that acts as a pro-survival or pro-death mechanism depending on different physiological or pathological conditions [20], is important for embryonic development, neural proliferation, and differentiation during brain development [21, 22]. Tight junctions are essential for establishing the selectively permeable barrier between neighboring cells, tight junctions formed between brain capillary endothelial cells help to create a blood-brain barrier that acts to protect the brain from harmful substances and maintain brain homeostasis, and modified tight junction protein expression and disrupted blood-brain barrier structure are observed in cerebral pathologies [23, 24]. Calcium signaling regulates nearly every aspect of neural development, including neural induction, neural rosettes formation, neural progenitor cell proliferation, neuronal migration, and differentiation [25–27], and specific synaptic connection development and impairments in calcium-dependent network maturation are associated with neurodevelopment disorders [28].

Additionally, DMGs altered by maternal FA deficiency were enriched distinctively in mTOR signaling pathway, Notch signaling pathway, neurotrophin signaling pathway, and protein processing in ER. The mTOR signaling plays vital roles in dendritic formation and axon guidance during normal brain development [29], and aberrant mTOR signaling has been implicated in neurodevelopment disorders characterized by cognitive deficits, such as tuberous sclerosis and fragile X syndrome [30]. Notch signaling maintains the neural stem cell pool during brain development [31], regulates cortical cell-type differentiation in an orderly progression [32], and interacts with Reelin signaling regulating cortical neuronal migration [33]. Neurotrophins are growth factors consisting of nerve growth factor, brain-derived neurotrophic factor, neurotrophin (NT) 3, and NT-4, which participated in a wide aspect of neurodevelopment, including neural survival, proliferation, differentiation, myelination, apoptosis, and axonal growth [34]. The ER is a subcellular organelle where proteins are folded with the help of lumenal chaperones. Proteins that correctly folded are packaged into transport vesicles that shuttle to Golgi complex, while the accumulation of unfolded or misfolded proteins in the ER lumen triggered ER stress and unfolded protein response that may induce aberrant neuronal differentiation and inhibition of dendrite outgrowth [35].

While DMGs altered by maternal FA supplementation were enriched distinctively in MAPK signaling pathway, TGF-β signaling pathway, and axon guidance, MAPKs have been involved in the modulation of hippocampal development. An extracellular signal-regulated kinase (ERK) signaling regulates the maintenance of neural progenitor cells in the dentate gyrus [36]. ERK and p38 activities are dynamically regulated during postnatal development that may correlate with the occurrence of hippocampal programmed cell death and synaptogenesis [37]. TGF-β signaling has been implicated in axonal formation and specification and neuronal migration during brain development [38, 39]. Axon guidance, including axon outgrowth, axon repulsion, and axon attraction, represent critical stages in neuronal network formation.

### Potential mechanisms of maternal FA intake during pregnancy impacting offspring’s long-term learning/memory abilities through DNA methylation

DNA methylation on cytosine residues in CpG sequences is a mitotically stable epigenetic modification that can produce long-term changes in gene expression [11, 40], and transient inhibition of DNA methylation during development exerts long-term deleterious effects on synaptic plasticity and memory abilities in adult mice [14]. Consistently, the results in the present study showed that the DMGs altered by maternal FA deficient and supplementation were both enriched in pathways of cGMP-PKG signaling pathway, gap junction, and calcium signaling pathway. cGMP-PKG signaling mediates early memory consolidation and early-phase LTP [41]. In addition, NO-cGMP-PKG signaling pathway regulates fear memory consolidation by promoting both presynaptic and postsynaptic alterations following fear conditioning at lateral amygdala synapses via NO-driven “retrograde signaling” [42]. Gap junctions in the mammalian brain act to synchronize neuronal activity and connect glial cells participating in the regulation of brain metabolism and homeostasis and suggested to contribute to fear learning and memory [43, 44]. Postsynaptic calcium signaling is suggested to induce activity-dependent LTP in CA1 and CA2 hippocampal neurons; calcium/calmodulin may activate many plasticity-inducing pathways, such as CaMK, Ras/ERK, and PKA [45], and astroglial calcium signaling displays short-term plasticity and downregulates synaptic transmission [46].

Additionally, DMGs altered by maternal FA deficiency were enriched distinctively in mTOR signaling pathway, Rap1 signaling pathway, Notch signaling pathway, endocytosis, and neurotrophin signaling pathway. mTOR regulates protein translation in response to neuronal activity, thereby modulating synaptic plasticity and long-term memory formation. It has been implicated in fear of memory acquisition/consolidation in the hippocampus/amygdala [47]. Small GTPase Rap1 signals synaptic glutamate receptor (GluR)-2/3 AMPARs removes by activating Rap1-p38MAPK signaling during LTD [48]. Notch signaling plays a critical role in brain development. Recently, emerging evidence has suggested that Notch signaling also plays a vital role in synaptic plasticity and spatial memory formation [49, 50]. Neurotrophins are firstly described as growth factors that support neuronal differentiation and survival. Later studies have indicated their roles in synapse formation and plasticity, including increasing neurotransmitter output, membrane excitability, and synaptic contacting size locally at synapse and affecting gene expression and phenotypic enhancement distally at the neuronal soma [51].

While DMGs altered by maternal FA supplementation were enriched distinctively in MAPK signaling pathway, ErbB signaling pathway, glutamatergic synapse, and neuroactive ligand-receptor interaction, MAPK is activated after learning, or LTP induction in the hippocampus and amygdala, and pharmacological inhibition of MAPK activation impairs LTP, learning, and long-term memory [52]. ErbB signaling modulates hippocampal metabotropic GluRI-dependent LTD and object recognition memory [53], and pharmacological inhibition of ErbB signaling during adolescence impairs reference memory in adult mice [54]. In the glutamatergic synapse, glutamate released from neuron presynaptic terminal acts on postsynaptic ionotropic or metabotropic GluRs to regulate synaptic plasticity and, afterward, is removed from the synaptic cleft by EAATs located either on the presynaptic terminal, postsynaptic neuron, or neighboring glial cells. Signals resulting from ligand-receptor interactions trigger intracellular signaling that ultimately modulates gene expression, which could be the underlying basis of learning and memory [55].

## Conclusion

In conclusion, the present study found that maternal FA deficiency and supplementation during pregnancy altered DNA methylation profiles in brain tissue of male offspring, and those DMGs were implicated in neurodevelopment and learning/memory abilities. These findings provide novel insights into the mechanism studies of maternal FA intake impacting offspring’s neurodevelopment and learning/memory abilities with DNA methylation involved. Validation studies and more in-depth discussion in the mechanisms of FA deficiency or FA-supplemented conditions during pregnancy will be the focus of further studies.

## Data Availability

All of the data are available with reasonable request from the corresponding author.

## Abbreviations

DEPs: Differential enrichment peaks
DMGs: Differentially methylated genes
DNMT: DNA methyltransferases
ER: Endoplasmic reticulum
FA: Folic acid
GluR: Glutamate receptor
GO: Gene ontology
HCP: High-CpG-density promoter
ICP: Intermediate-CpG-density promoter
LCP: Low-CpG-density promoter
LTD: Long-term depression
LTP: Long-term potentiation
MAPK: Mitogen-activated protein kinase
MeDIP: Methylated DNA immunoprecipitation
mTOR: Mammalian/mechanistic target of rapamycin
TGF: Transforming growth factor
TSSs: Transcription start sites
